# Novel avian single-chain fragment variable (scFv) targets dietary gluten and related natural grain prolamins, toxic entities of celiac disease

**DOI:** 10.1186/s12896-015-0223-z

**Published:** 2015-12-01

**Authors:** Valerie Stadlmann, Hanna Harant, Irina Korschineck, Marcela Hermann, Florian Forster, Albert Missbichler

**Affiliations:** Sciotec Diagnostics Technologies GmbH, Tulln, Austria; Ingenetix GmbH, Vienna, Austria; Department of Medical Biochemistry, Division of Molecular Genetics, Medical University of Vienna, Vienna, Austria

**Keywords:** Celiac disease, Celiac disease treatment, Wheat, Gliadin, Dietary gluten, Prolamins, scFv

## Abstract

**Background:**

Celiac disease (CD) is a chronic, small intestinal inflammatory disease mediated by dietary gluten and related prolamins. The only current therapeutic option is maintenance of a strict life-long gluten-free diet, which implies substantial burden for CD patients. Different treatment regimes might be feasible, including masking of toxic celiac peptides with blocking antibodies or fragments thereof. The objective of this study was therefore to select and produce a recombinant avian single-chain fragment variable (scFv) directed against peptic-tryptic digested gliadin (PT-Gliadin) and related celiac toxic entities.

**Results:**

Gluten-free raised chicken of same age were immunized with PT-Gliadin. Chicken splenic lymphocytes, selected with antigen-coated magnetic beads, served as RNA source for the generation of cDNA. Chicken V_H_ and V_L_ genes were amplified from the cDNA by PCR to generate full-length scFv constructs consisting of V_H_ and V_L_ fragments joined by a linker sequence. ScFv constructs were ligated in a prokaryotic expression vector, which provides a C-terminal hexahistidine tag.

ScFvs from several bacterial clones were expressed in soluble form and crude cell lysates screened for binding to PT-Gliadin by ELISA. We identified an enriched scFv motif, which showed reactivity to PT-Gliadin. One selected scFv candidate was expressed and purified to homogeneity. Polyclonal anti-PT-Gliadin IgY, purified from egg yolk of immunized chicken, served as control. ScFv binds in a dose-dependent manner to PT-Gliadin, comparable to IgY. Furthermore, IgY competitively displaces scFv from PT-Gliadin and natural wheat flour digest, indicating a common epitope of scFv and IgY. ScFv was tested for reactivity to different gastric digested dietary grain flours. ScFv detects common and khorasan wheat comparably with binding affinities in the high nanomolar range, while rye is detected to a lesser extent. Notably, barley and cereals which are part of the gluten-free diet, like corn and rice, are not detected by scFv. Similarly, the pseudo-grain amaranth, used as gluten-free alternative, is not targeted by scFv. This data indicate that scFv specifically recognizes toxic cereal peptides relevant in CD.

**Conclusion:**

ScFv can be of benefit for future CD treatment regimes.

## Background

Celiac disease (CD) is a chronic, small intestinal, immune-mediated disease driven by dietary wheat gluten and related prolamins in rye and barley [1, 2]. Disease hallmarks are varying degrees of villous atrophy, crypt hyperplasia, intraepithelial lymphocyte infiltrates and serum auto-antibodies [3]. Although the hellenic physician Artaeus the Cappadocianin already described CD symptoms in the second century AD [4], it was not until the 1940s when the link to dietary prolamins was established by the Dutch pediatrician Dicke [5, 6]. The current definition of CD is based on the 14th International CD Symposium in 2011 which led to ’The Oslo definitions for celiac disease and related terms’, published by Ludvigsson et al. in 2013 [6]. Despite all efforts to describe and understand disease mechanisms, there is still lack of appropriate treatment. A stringent, life-long gluten free diet (GFD) is the only option, favoring abatement of symptoms and improvement of intestinal barrier function in most CD patients. Some potential treatment agents are already in preclinical and clinical phases, for example glutenase ALV003 [7, 8] (clinicaltrials.gov identifier NCT00959114, NCT01255696) or tight junction regulator larazotide acetate AT-1001 [9] (clinicaltrials.gov identifier NCT01396213, NCT00620451). Another potential approach for CD treatment could be the blocking of toxic dietary peptides by antibodies or fragments thereof, which was the objective of this study.

Wheat gluten peptides, subdivided dependent on solubility in watery alcohols into soluble gliadins and insoluble glutenins, represent a heterogeneous mix of proteins of different molecular weights [10]. Peptic, tryptic digests of the gliadin fraction, termed PT-Gliadin, mimic the peptide fraction entering the duodenum after gastric digestion. PT-Gliadin is a commonly chosen model antigen for studying CD [11–13] and served therefore as immunogen for chicken in this study. We produced PT-Gliadin reactive chicken yolk antibodies (IgY) and used immunized chicken as source for the production of recombinant antibody fragments in the single-chain format. Chicken IgY represents the avian equivalent to mammalian IgG, though IgY has many advantages considering human applications: IgY exerts no mammalian complement activation, rheumatoid factor interaction or cross-reactivity with mammalian IgG [14]. IgY can be easily purified from yolks of immunized chicken by precipitation [15] or chromatography methods [16]. In principle, IgY can be administered orally in enteric coated form [17, 18]. Chicken IgY is described for different diagnostic and therapeutic applications [19–22], including anti-Gliadin IgY for CD treatment [23]. However, we consider the use of yolk IgY inefficient for clinical large scale production. Thus our goal was to engineer IgY fragments in a recombinant format, which can be expressed in *Escherichia coli (E. coli)* in soluble form and offers a scalable production process. In this study we report the cloning and selection of an avian single-chain fragment variable (scFv) directed against PT-Gliadin. We present data demonstrating the in vitro potential of scFv in targeting PT-Gliadin and natural flour digests. We observed comparable binding characteristics for scFv and polyclonal yolk IgY.

## Methods

### Preparation of PT-Gliadin

PT-Gliadin was prepared from wheat gliadin (Sigma) according to previously described methods [24] with some adjustments. Briefly, 10 g gliadin (gliadin from wheat, Sigma-Aldrich) was subjected to 40 ml 20 mM sodium acetate buffer, pH 4.5. 800 μl immobilized pepsin (Thermo Scientific), washed three times with sodium acetate buffer according to manufacturer’s instruction, was added to the gliadin-buffer mixture. Peptic digest was performed by overnight incubation at 37 °C with agitation at 350 rpm. Pepsin was separated by centrifugation at 4000 x g for 2 min and aspiration of the supernatant. Pepsin was regenerated and stored according to manufacturer’s instruction. The supernatant was adjusted to pH 8 with 1 N NaOH. 800 μl immobilized trypsin (Thermo Scientific), washed three times with 20 mM ammonium hydrogen carbonate according to manufacturer’s instruction, was added to the gliadin digest. Tryptic digest was performed by overnight incubation at 37 °C with agitation. The volume was adjusted with ammonium hydrogen carbonate to 45 ml and the mixture incubated for further 3 h at 37 °C. Trypsin was separated by centrifugation at 4000 x g for 2 min and aspiration of the supernatant. Trypsin was regenerated and stored according to manufacturer’s instruction. The supernatant (containing PT-Gliadin) was filtrated through fluted and subsequently through 0.45 μm syringe filters. Total protein content was measured by BCA test (Pierce™ BCA Protein Assay Kit, Thermo Scientific) and PT-Gliadin was lyophilized to equal protein amounts (~8 mg/ml) and stored at 4 °C. When needed, PT-Gliadin was resuspended in 1 ml sterile Tris buffered saline (TBS, made from 10 x concentrate, Sigma) and total protein content was confirmed by BCA measurement. For the immunization of chicken, PT-Gliadin was resuspended in 10 % acetic acid.

### Preparation of flour digests

100 mg NaCl (Sigma-Aldrich) and 160 mg pepsin were dissolved in 25 ml H_2_O, pH was adjusted to 1.2 with 1 M HCl and volume was adjusted to 50 ml with H_2_O. This solution mimics gastric digestion and is referred to as simulated gastric fluid (SGF) according to United States Pharmacopoeia (USP32-NF27).

Barley (Rollgerste Gerstengraupen, Alnatura) and amaranth (Bio Amaranth “Das Inka-Korn”, HOLO) grains were grinded with mortar and pestle and 1 g of the grist was subjected to 5 ml SGF according to the protocol for flours described below. Wheat flour (Bio Weizen Vollkornmehl, Ja! Natürlich), khorasan wheat flour (Bio Kamutmehl, Vollkraft), rye flour (Bio Roggen Vollkornmehl, Rosenfellner Mühle), rice flour (Bio Reismehl fein gemahlen, HOLO), and corn flour (Polenta, Fini’s Feinstes,) were subjected directly to SGF: 5 ml SGF was added to 1 g flour or grist and incubated for 1 h at 37 °C with agitation at 350 rpm. Pepsin was separated by centrifugation at 14,800 x g for 4 min and aspiration of the supernatant. Pepsin was regenerated and stored according to manufacturer’s instructions. Supernatant (resembling the flour digest) was adjusted to pH 8 with 1 N NaOH and the end volume was adjusted to 8 ml.

### Immunization of laying hens

Brown laying hens, *Gallus gallus domesticus* Tetra SL, were raised on a gluten free diet from day 1 after hatching. Hens were primary immunized at an age of eight weeks by injecting a mixture of 250 μg PT-Gliadin (dissolved in 10 % acetic acid) and adjuvant into the pectoral muscle. Booster immunizations were given in intervals of three to four weeks. Blood samples were drawn pre and in weekly intervals after immunization and stored in 10 % EDTA at – 20 °C until analysis. All animal procedures followed the institutional laboratory animal research guidelines and were approved by the State veterinary and food administration of the Slovak republic.

### Anti-PT-Gliadin-IgY

60 eggs collected from various immunized chicken housed in the same facility were pooled to an IgY batch. IgY fraction was precipitated from egg yolks according to a previously developed in-house method: Yolks were separated from egg white and mixed with the 9-fold amount of deionized H_2_O with respect to yolk weight. 3 ml caprylic acid, pH 4.0 (Merck), per egg yolk was added drop-wise and the solution stirred for 1 h. Solution was filtered through fluted filter and washed with 30 ml deionized-H_2_O per egg yolk. Filter cake was discarded and the pH of the solution was adjusted to 7.2 with 1 N NaOH. 20 % (v/v) pre-chilled ethanol (Ethanol absolute, Roth) was added slowly under constant stirring at 400 rpm (volume ethanol represent ¼ of volume caprylic acid precipitation). Solution was precipitated over night in centrifuge beakers at −10 °C, followed by a centrifugation step at 0 °C and 11,000 x g for 30 min. Supernatant was discarded, pellet was dissolved in 15 ml phosphate buffer saline (PBS) and stored at −20 °C until use. 12 ml of the resuspended solution was subjected to 12 ml 10 mM phosphate buffer supplemented with 100 mM NaCl. 3.5 % (w/v) polyethylene glycol 6000 (PEG, Sigma-Aldrich) was added and mixture stirred at 400 rpm for 30 min at room temperature. Solution was centrifuged at 13,000 x g for 10 min and the supernatant was filtered through a fluted filter. 12 % (w/v) fresh PEG 6000 was added and the mixture stirred at 400 rpm for 1 h at room temperature. Solution was centrifuged at 13,000 x g for 10 min. The supernatant was discarded and the pellet was resuspended in a total volume of 12 ml 10 mM phosphate buffer supplemented with 100 mM NaCl. 12 % (w/v) fresh PEG 6000 was added and the mixture stirred at 400 rpm for 1 h at room temperature. Solution was centrifuged at 13,000 x g for 10 min. The supernatant was discarded and the centrifugation step, discarding the supernatant, repeated twice. The pellet containing the purified IgY fraction was resuspended in TBS and stored at 4 °C until use.

### Cloning strategy for the generation of chicken scFvs

10–14 days after the third immunization, chicken with confirmed blood titer were sacrificed. Spleens were harvested and merged through a 100 μm nylon cell strainer (BD Biosciences) with 10 ml sterile phosphate buffered saline (PBS sterile, Life Technologies). 10 ml sterile ficoll solution (GE Healthcare) was pipetted underneath the cell suspension and tubes were centrifuged at 660 x g for 20 min for the formation of a density gradient. The interphase, containing mononuclear cells (lymphocytes and monocytes, respectively) was isolated, and the remaining gradient discarded. Mononuclear cells were washed with 1 ml fresh PBS, mixed be inverting the tube and centrifuged at 300 x g for 5 min. Supernatant was discarded and the washing step was repeated twice. Washed cells were diluted in PBS to a concentration of 1 x 10^7 cells/ml and afterward subjected to magnetic beads (Dynabeads M-450 Epoxy, Life Technologies), pre-coated with PT-Gliadin according to manufacturer’s instructions. 25 μl of pre-coated beads were washed according to protocol and added to 1 ml cell suspension. Suspension was incubated at 4 °C with end-over-end tilting for 20 min. Suspension was placed on a magnetic stand for 2 min, supernatant was discarded. Beads bound cells were washed six times with fresh, sterile PBS. PBS was discarded and beads bound cells were resuspended in 1 ml PBS. Beads bound cells were subjected immediately to total RNA extraction using ReliaPrep™ RNA Cell Miniprep System from Promega according to manufacturer’s instruction. Beads were separated magnetically prior to loading of the suspension on the RNA capturing mini column. RNA quantity was analyzed by NanoDrop 2000c spectrophotometer (Peqlab biotechnology GMBH). Complementary DNA (cDNA) was synthesized from ~1 μg total RNA using random hexameric primers (Life technologies) and SuperScript®III First-Strand Synthesis System (Life technologies) according to manufacturer’s instructions. cDNA was amplified using a set of specific primers for chicken V_H_ (forward: 5′-GCC GTG ACG TTG GAC GA-3′; reverse: 5′-GGA GGA GAC GAT GAC TTC GG-3′) and V_L_ (forward: 5′-AGG CTG ACT CAG CCG T-3′; reverse: 5′-ACC TAG GAC GGT CAG GG-3′) gene regions. Gel purified PCR products were subjected to a second PCR reaction, to introduce a peptide linker (GSTSGSGKPGSGEGSTKG) sequence [25] and restriction sites:

V_H_ gene products were amplified with primer sets to introduce *Nco*I/*Bam*HI sites (forward: 5′-ATG TCT CTA T**CC ATG G**CC GTG ACG TTG GAC GA-3′; *Nco*I site in boldface; reverse:

5′-ATG ATG **GGA TCC** GGG CTT GCC GCT ACC GGA AGT AGA GCC GGA GGA GAC GAT GAC TTC GG-3′, *Bam*HI site in boldface;). V_L_ gene products were amplified with primer sets to insert *Bgl*II and *Not*I sites (forward: 5′- CAT CAT **AGA TCT** GGT GAA GGT AGC ACT AAA GGT GCG CTG ACT CAG CCG T-3′, *Bgl*II site in boldface; reverse: 5′- GTG GTG GTG CTC TCG AGT **GCG GCC GC**G GGA CCT AGG ACG GTC AGG G-3′, *Not*I site in boldface). V_H_ and V_L_ re-amplification products were directly used for cloning into pCR® 2.1 by TOPO cloning (TOPO®^,^ Life technologies) according to manufacturer’s recommendation. 50 μl of chemically competent *Escherichia coli (E. coli)* One Shot® TOP10 (Life technologies) were transformed with 2.5 μl of TOPO V_H_ or V_L_ cloning products, respectively. Bacterial clones were grown overnight in 2 ml LB broth (Sigma) supplemented with 50 μg/ml ampicillin at 225 rpm at room temperature. Plasmid DNA from overnight cultures was isolated using Pure Yield™ Plasmid Mini Prep System from Promega. Plasmid DNA was subjected to sequencing using M13_rev sequencing primer (5′-CAG GAA ACA GCT ATG AC-3′) at Microsynth AG, Vienna, Austria. Sequencing analysis ensured the correct insertion of V_H_/V_L_ cloning products; at least four clones per plate were analyzed. To generate separate chicken V_H_ and V_L_ libraries, TOPO V_H_ or V_L_ cultures were scraped off the plates and transferred to 100 ml fresh LB broth (supplemented with 50 μg/ml ampicillin) each. Library cultures were incubated overnight at 225 rpm and 37 °C. Plasmid DNA of V_H_ and V_L_ library cultures was isolated with Pure Yield™ Plasmid Midiprep System from Promega. DNA concentration was quantified photometrical (BioPhotometer, dsDNA method, Eppendorf). V_H_ Plasmid DNA (corresponding to 5–10 μg DNA) was subjected to digestion with *Nco*I and BamHI (New England BioLabs), while V_L_ Plasmid DNA was digested with *Not*I and *Bgl*II (New England BioLabs). The plasmid vector pET28a(+)(Novagen) was digested with *Nco*I and *Not*I, followed by dephopsphorylation with alkaline phoshatase (Roche)*.* Restriction digests were separated by preparative 1.2 % agarose gels and V_H_ and V_L_ bands isolated from the gel with sterile scalpels and purified using QIAEX II Gel Extraction Kit (Qiagen). A 3-way ligation of V_H_ and V_L_ fragments and pET28a(+) expression vector (carrying a His_6_-tag) was performed to generate full-length scFvs: *E. coli* One Shot® BL21(DE3) (Life technologies) were transformed with the ligation products. As quality control, at least four clones per plate were cultivated overnight as mentioned above and analyzed by sequencing with pET28a_seq forward (5′-GTC CGG CGT AGA GGA TCG-3′) and reverse (5′-ATC CGG ATA TAG TTC CTC CTT T-3′) primers, to ensure the insertion of correct full-length scFvs in-frame with the C-terminal hexahistidine tag.

### Soluble expression of His_6_-tagged-scFvs in E. coli BL21 (DE3)

Individual bacterial clones were cultivated in LB supplemented with 25 μg/ml of kanamycin (kanamycin sulfate, Sigma-Aldrich) with agitation at 225 rpm over night at room temperature. The next day, fresh LB medium (supplemented with 25 μg/ml of kanamycin) was inoculated with 1/10 volume of overnight culture. Expression cultures were cultivated at room temperature with agitation at 225 rpm until optical density (OD)_600_ reached 0.4 – 0.5. Protein expression was induced by the addition of 1 mM sterile isopropyl ß-D-1-thiogalactopyranoside (IPTG, Sigma-Aldrich) to expression cultures. At selected time points pre- and post induction, cell pellets were harvested by centrifugation at 2000 x g for 20 min. Supernatants were discarded and cell pellets stored frozen at −80 °C until cellular lysis. Cell pellets were lysed with Qproteome™ Bacterial Protein Prep Kit from Qiagen according to manufacturer’s instruction. The soluble supernatants, referred to as crude extracts, were stored at −20 °C.

### Purification of anti-PT-Gliadin scFv

Small-scale purification of bacterial crude extracts was performed using Ni-NTA-spin columns (Thermo Scientific: 0.2 ml or 10 ml volumes respectively) according to manufacturer’s instructions. Eluates were buffer exchanged in TBS using Zeba Spin Desalting Columns (Thermo Scientific, 7 K MWCO, 0.5 ml or 10 ml volumes respectively) according to manufacturer’s instructions. Purified proteins were stored at −20 °C. For large scale expression, scFv was cultivated in LB supplemented with 25 μg/ml of kanamycin in lab fermenter scale (1–5 l culture volume respectively). Protein expression was induced with 1 mM IPTG and cell pellets were harvested 4–5 h post induction by centrifugation at 2000 x g for 20 min. Cell pellets were resuspended in 10 mM imidazole buffer (3–4 buffer excess in relation to wet biomass). Solution was treated three times for 30 s with an ultra-turaxx on ice. Solution was homogenized for 5 min 15 s at 100–150 MPa on ice (for 20 ml volumes respectively). Homogenizates, containing soluble ScFv, were purified by Immobilized Metal Affinity Chromatography (IMAC) with HiTrap TALON crude columns (1 or 25 ml size, Co^2+^) and imidazole (10 mM imidazole in application buffer, 500 mM imidazole in elution buffer) using an äkta purifier (Äkta pure, GE Healthcare). IMAC eluates were further purified by Size-Exclusion-Chromatography (Superdex 75 pg, M_r_ 3 000 to 70 000. 16/600, GE Healthcare) using an äkta purifier (Äkta pure, GE Healthcare). Pooled eluates were buffer exchanged into TBS via spin columns (Amicon® Ultra-4 Centrifugal Filter Units) and stored at −20 °C. ScFv of size-exclusion chromatography purity grade was used directly for ELISA and Western Blot characterization methods.

### Plate coating

For comparison of PT-Gliadin and different grain digests, 96-well plates (biomat HB) were coated with 100 μl/well of PT-Gliadin dilution ranges (1000, 5000, 250, 125 ng/ml respectively); or digests of wheat, khorasan wheat (kamut), rye, barley, corn, rice and amaranth flours (15.0, 7.5, 3.8, 1.9 μg/ml respectively). For all other ELISA experiments, 96-well plates were coated with constant concentrations (1000 ng/ml PT-Gliadin or 15 μg/ml of wheat flour, respectively). Plates were coated with antigens diluted in 20 mM Na_2_CO_3_ buffer, pH 9.6 over night at 4 °C. Plates were blocked with 300 μl per well of 1 % (w/v) PEG 6000 in 20 mM Na_2_CO_3_ for 1 h. Control plates were treated in the same way with the exception that antigen was omitted. Plates were dried and stored at room temperature in a dry atmosphere.

### Direct ELISA

All sample and antibody dilutions were performed in TBST buffer (TBS with 0.05 % (v/v) Tween 20) supplemented with 1 % (v/v) BSA (Albumin solution from bovine serum, 30 % in 0.85 % sodium chloride, Sigma-Aldrich): EDTA-plasma samples of pre-immune and immunized chicken were diluted 1000-fold. IgY or scFv (size-exclusion chromatography purity grade) were diluted to 1000, 500, 250, 125, 63, 31 ng/ml.

Samples were added in triplicates (100 μl per well) to coated and control wells. Plates were incubated for 1 h at room temperature with agitation at 350 rpm. Plates were washed four times with 300 μl per well of TBST. 100 μl per well of goat anti-chicken-IgG (H + L)-HRP antibody (southern biotech) was added at a 1:3000 dilution. Plates were incubated for 1 h at room temperature with agitation at 350 rpm. Plates were washed four times with 300 μl per well of TBST. 100 μl pre-mixed tetramethybenzidine (TMB) substrate (Pierce) was added per well and developing color reaction was stopped with 50 μl of 2 N H_2_SO_4_. Absorbance was measured at 450 nm in a micro plate reader (GloMax-Multi Detection system, Promega).

For Screening of scFv candidates, bacterial crude extracts were diluted 10-fold and 50 μl per well was subjected to coated and control wells, respectively. Plates were incubated for 1 h at room temperature with agitation at 350 rpm. Mouse anti-penta-His antibody (Qiagen) was diluted 250-fold and 50 ng per well was added to the crude extract dilutions. Plates were incubated at 350 rpm for 2 h at room temperature. Plates were washed four times with TBST. Goat anti-mouse-IgG (H + L)-HRP antibody (southern-biotech) was diluted 1000-fold and 100 ng per well was added. Plates were incubated at 350 rpm for 1.5 h at room temperature. Plates were washed four times with 300 μl per well TBST and ELISA was developed as mentioned above.

### Competitive ELISA

All sample and antibody dilutions were performed in TBST buffer supplemented with 1 % (v/v) BSA.

50 μl per well of Anti-PT-Gliadin IgY at concentrations of ~ 100, 400, 800, 1600 and 3200 μg/ml were added in triplicates to coated or control wells. Plates were incubated for 1 h at room temperature with agitation at 350 rpm. 50 μl of scFv was added per well in a ~ 0.04 [μg/ml] concentration. Plates were incubated for 40 min at room temperature with agitation at 350 rpm. Plates were washed four times with 300 μl per well TBST. 100 μl per well of mouse anti-penta-His antibody was added at a 1:500 dilution. Plates were incubated for 1 h at room temperature with agitation at 350 rpm and washed four times with 300 μl per well TBST. 100 μl per well of goat anti-mouse-IgG-HRP was added in a 1:1000 dilution and plates were incubated for 1 h at room temperature with agitation at 350 rpm. Plates were washed four times with TBST. ELISA was developed according to direct ELISA.

### Western blot

30 μg per lane crude bacterial extracts were separated by SDS-PAGE (NuPage 12 % Bis Tris Gel, Life technologies) using EI9001-XCELL II Mini-Cell (Life technologies). Proteins were electrophoretically transferred (Trans-Blot® SD Semi-Dry Transfer Cell, Bio-Rad) to activated 0.45 μm PVDF membranes (Life Technologies). Unlike otherwise noted, all following incubation steps were performed at room temperature with agitation at 350 rpm: Membranes were blocked with 3 % (w/v) skim milk powder (Fluka) in TBS for 1 h. Membranes were incubated simultaneously with mouse-anti-penta-His antibody (Qiagen) at a 1:500 dilution and mouse-anti-HSP60 (Heat Shock Protein 60, Mouse monoclonal antibody, Thermo Scientific) at a 1:7500 dilution in TBST supplemented with 1 % milk powder for 1 h. Membranes were washed twice for 10 min with TBST, followed by incubation in anti-mouse-IgG-HRP (Amersham ECL Western Blotting Reagent Pack, GE healthcare) at a 1:2000 dilution in TBST supplemented with 1 % milk powder for 1 h. Membranes were washed once in TBST and once in TBS. Immunoreactivity was assessed by incubating membranes in Western Blot substrate (SuperSignal® West Pico Chemiluminescent Substrate, Thermo Scientific) using the ECL chemiluminescence Western Blot system (Amersham Hyperfilm™ ECL, GE Healthcare).

10 μg per lane PT-Gliadin/ wheat flour digest or 50 μg per lane digests of wheat, khorasan wheat (kamut), rye, barley, corn rice and amaranth were separated by SDS-PAGE, transferred to PVDF membranes and blotted as described above. Membranes were incubated over night at +4 °C with scFv (size-exclusion chromatography purity grade) at a 1:500 dilution (~15 μg/ml), or 1:3000 dilution (~2.5 μg/l) or IgY at a 1:100000 dilution (~1 μg/ml) in TBST supplemented with 1 % (w/v) milk powder. Membranes were washed twice for 10 min with TBST two times followed by an incubation in goat anti-chicken-IgG(H + L)-HRP antibody (southern biotech) at a 1:12000 dilution in TBST supplemented with 1 % milk powder for 1 h. Membranes were washed once in TBST and once in TBS. Western Blot was developed as described above.

### Data evaluation & statistical analysis

ELISA data was analyzed using GraphPad Prism 6 Software. Unlike otherwise noted, data represent mean values of triplicates with standard error of the mean (SEM) shown as error bars. For determination of mean affinity constants, direct ELISA data was analyzed by 3-parameter curve-fit nonlinear regression, OD_50_ values were derived for each particular antigen coating concentration. The final mean affinity constant represents the mean value (+/−SEM) of 4 different calculations: 3 calculated affinity constants at antigen coating ratio of 2; 1 calculated affinity constants at antigen coating ratio of 8. Each single calculation of affinity constant was performed according to the following equation: K_aff_ = (n − 1)/2 (n[AB′]_t_ − [AB]_t_) [26, 27] for coating with two antigen concentrations [AG]t and [AG’]t and OD_50_ values at concentration [AB]_t_ and [AB’]_t_; *n* = [AG]_t_/[AG’]_t_ and corresponds to antigen coating ratio.

## Results

### Production of PT-Gliadin reactive chicken IgY

**Fig. 1:**
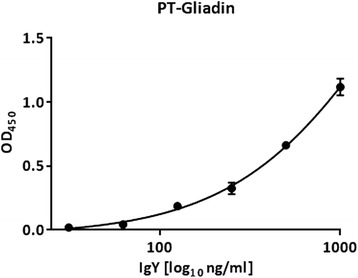
IgY binds to PT-Gliadin in a concentration -dependent manner. Purified yolk IgY was diluted in indicated concentrations and binding to PT-Gliadin was tested by ELISA as described in Methods. Data shown are OD (450 nm) values and represent mean values (+/− SEM) of triplicates

We previously developed an in-house method for the purification of IgY from yolks of immunized chicken, which is based on caprylic acid precipitation and subsequent PEG-purification. The resulting polyclonal yolk IgY was tested for binding to PT-Gliadin by ELISA. We were able to demonstrate concentration -dependent binding of chicken IgY to PT-Gliadin (Fig. 1). Yolk IgY served as our control for characterization of PT-Gliadin reactive scFv.

### Production and selection of PT-gliadin reactive scFv

**Fig. 2:**
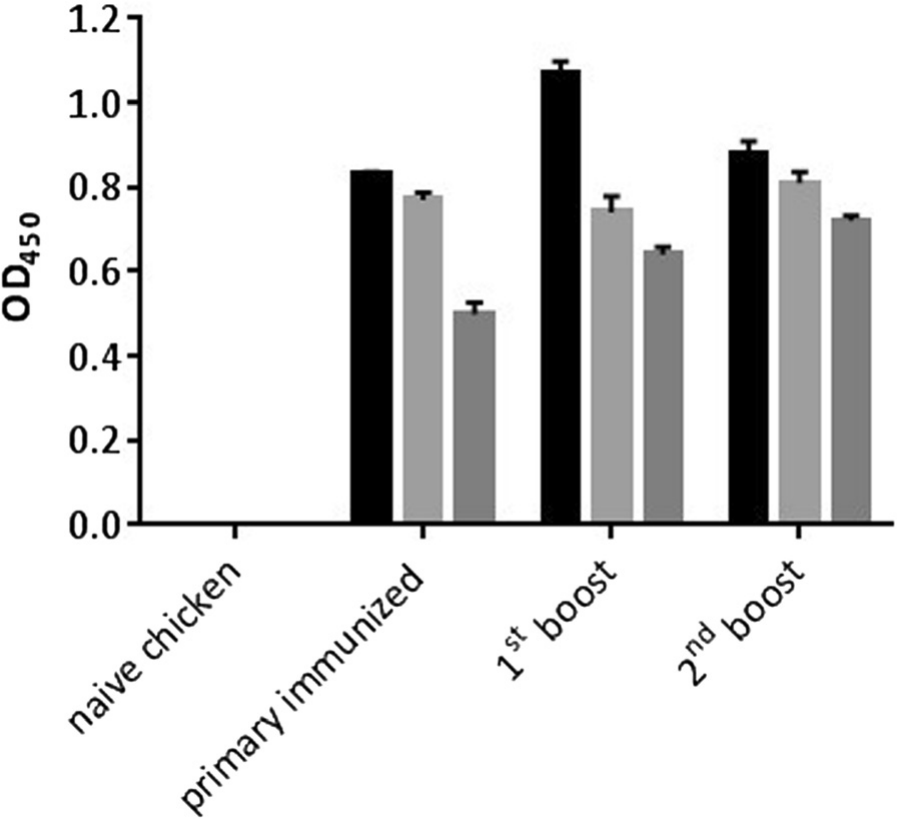
Anti-PT-Gliadin EDTA-plasma titer of gluten-free raised chicken. EDTA-plasma from naïve control chicken and chicken one week after primary, 1^st^ or 2^nd^ booster immunization was analyzed by ELISA as described in Methods. Data shown are OD (450 nm) values of three individual chicken displayed as mean values (+/− SEM) of triplicate measurements

**Fig. 3:**
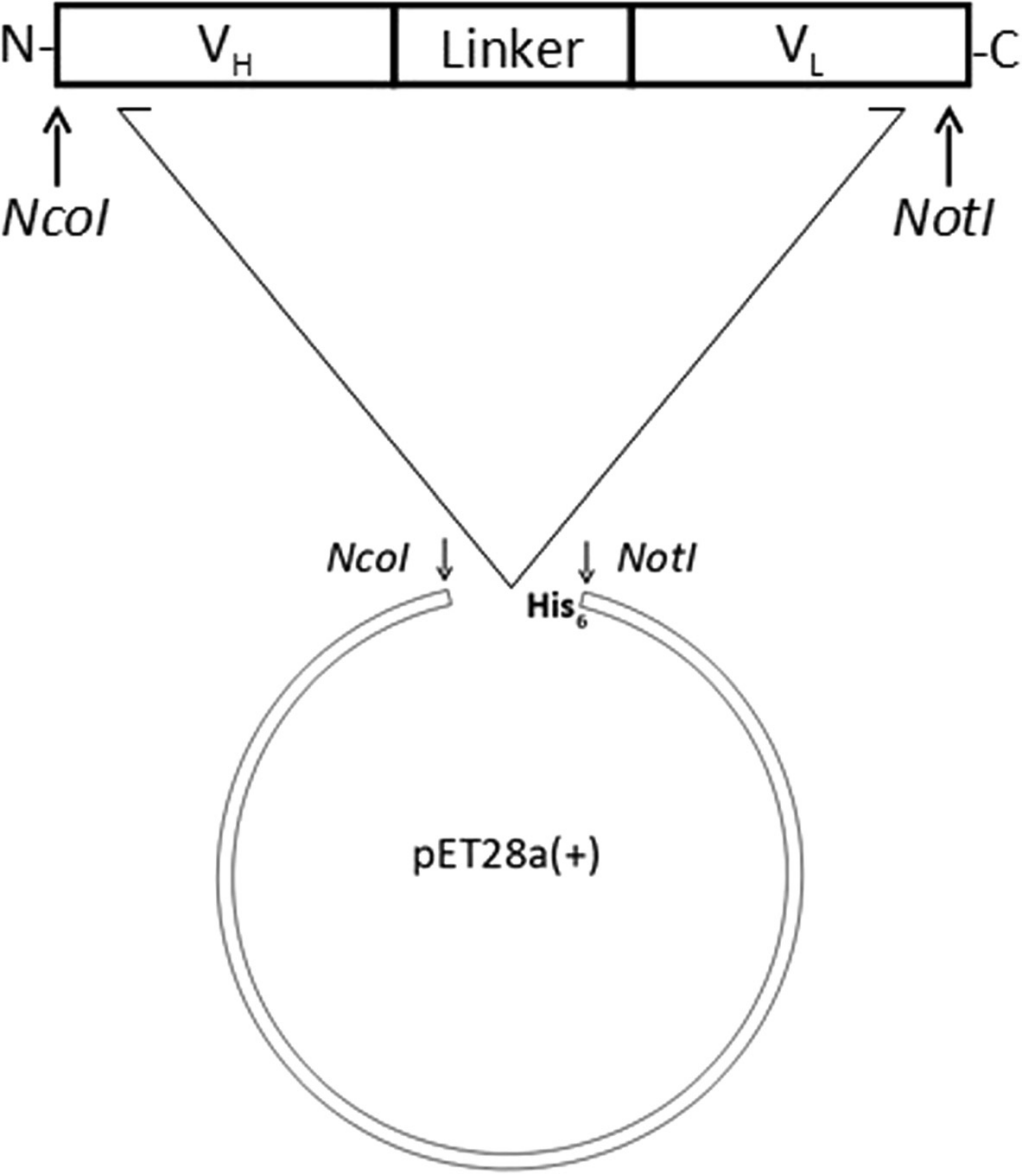
Schematic view of scFv fragment cloned into the expression vector pET28a (+). V_H_ and V_L_ fragments assemble to full-length scFv in between the linker peptide and are ligated into pET28a (+) vector, which provides a C-terminal hexahistidine tag

**Fig. 4:**
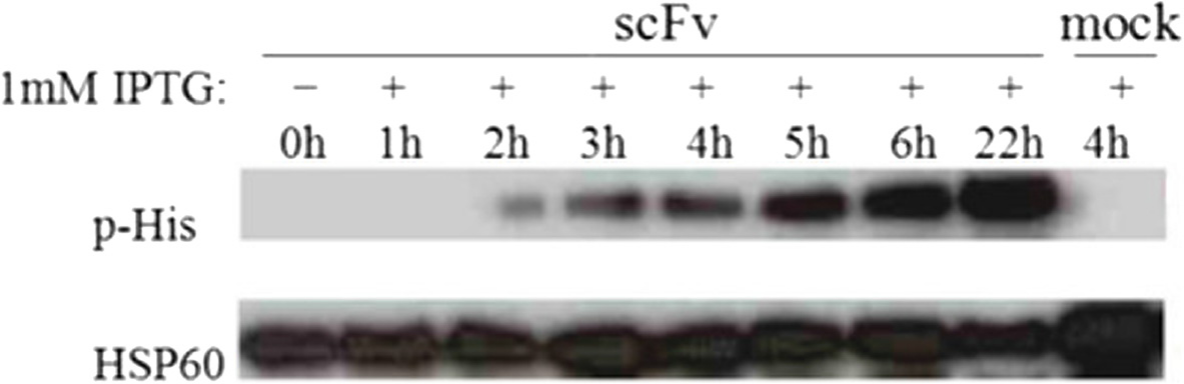
Expression time course of scFv which is soluble expressed in *E. coli* BL21/ (DE3). Crude extracts (30 μg protein per lane) from indicated time points before/after induction with 1 mM IPTG were separated by SDS-PAGE and blotted on PVDF membranes. Western Blot was performed as described in Methods. The upper lane shows target protein expression, the lower lane (*E. coli* 60 kDa HSP60 protein) represents the loading control. Mock represents the negative control (lysates of bacteria transformed with empty vector). Experiment shown is representative for at least three repeated experiments

For the production of PT-Gliadin reactive scFvs, we relied on chicken which were immunized three times and showed a respectable antibody blood titer (Fig. 2). Chicken were sacrificed 10–14 days after the third immunization. Splenic lymphocyte fractions were harvested by ficoll gradient centrifugation and mononuclear cells were subjected to magnetic beads previously coated with PT-gliadin. This step was applied to enrich antigen specific B lymphocytes which bind to PT-Gliadin, coated on beads. RNA of beads-bound cells was extracted and cDNA was synthesized. Chicken V_H_ and V_L_ genes were first amplified by PCR with specific primer pairs, followed by amplification in a second PCR step to introduce the linker sequence and sites for restriction digestion. These engineered V_H_ and V_L_ fragments were then ligated into plasmid vectors to generate V_H_ and V_L_ libraries, respectively. V_H_ and V_L_ fragments were excised from the plasmids and ligated into the prokaryotic expression vector pET28a(+), which provides a C-terminal hexahistidine tag (Fig. 3). Screening (at least four clones per plate) revealed that the majority of clones carried correct scFvs (data not shown). Soluble protein expression overtime was assessed by western blot (Fig. 4). ScFvs are soluble expressed in *E. coli* with increasing target protein concentration over time. The harvesting time point was set to 4–5 h post induction for screening of PT-Gliadin reactive scFvs.

**Fig. 5:**
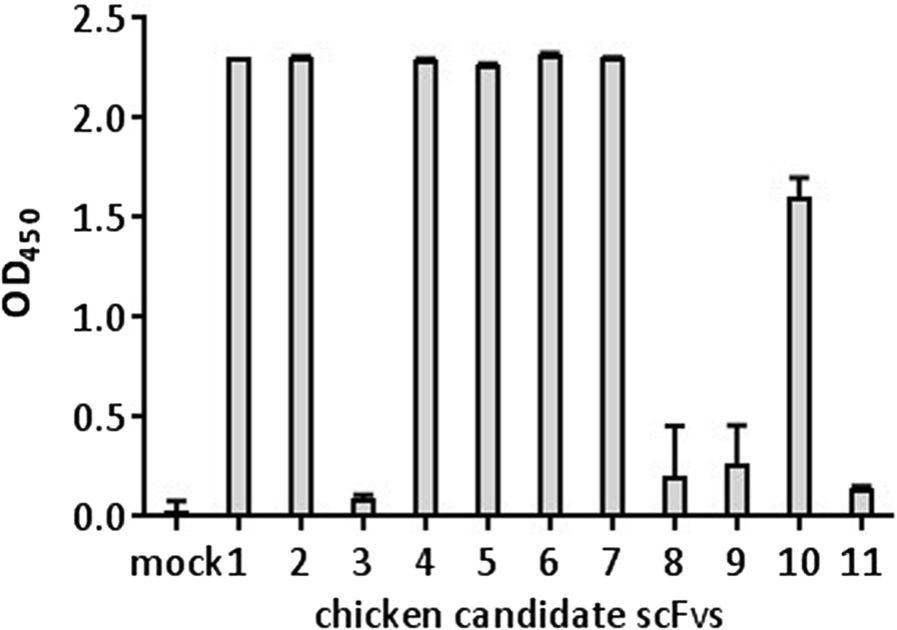
ELISA based testing of scFv candidates for reactivity to PT-Gliadin. Bacterial crude extracts expressing candidate scFvs were tested for antigen binding by ELISA as described in Methods. Samples were diluted 10-fold in TBS supplemented with 0.05 % Tween and 1 % BSA. Mock represents the negative control (crude extracts of bacteria transformed with empty vector). Data shown are OD (450 nm) levels and represent mean values (+/− SD) of duplicates

Crude extracts of up to 80 individual bacterial clones were tested for reactivity to PT-Gliadin by ELISA. Several crude extracts showed reactivity to PT-Gliadin (Fig. 5). Screening and protein alignment of these PT-Gliadin reactive scFvs revealed an enriched scFv motif. This scFv candidate was selected and further characterized in this study.

### Purified scFv candidate detects PT-Gliadin and peptic digested common whole wheat flour in vitro

**Fig. 6:**
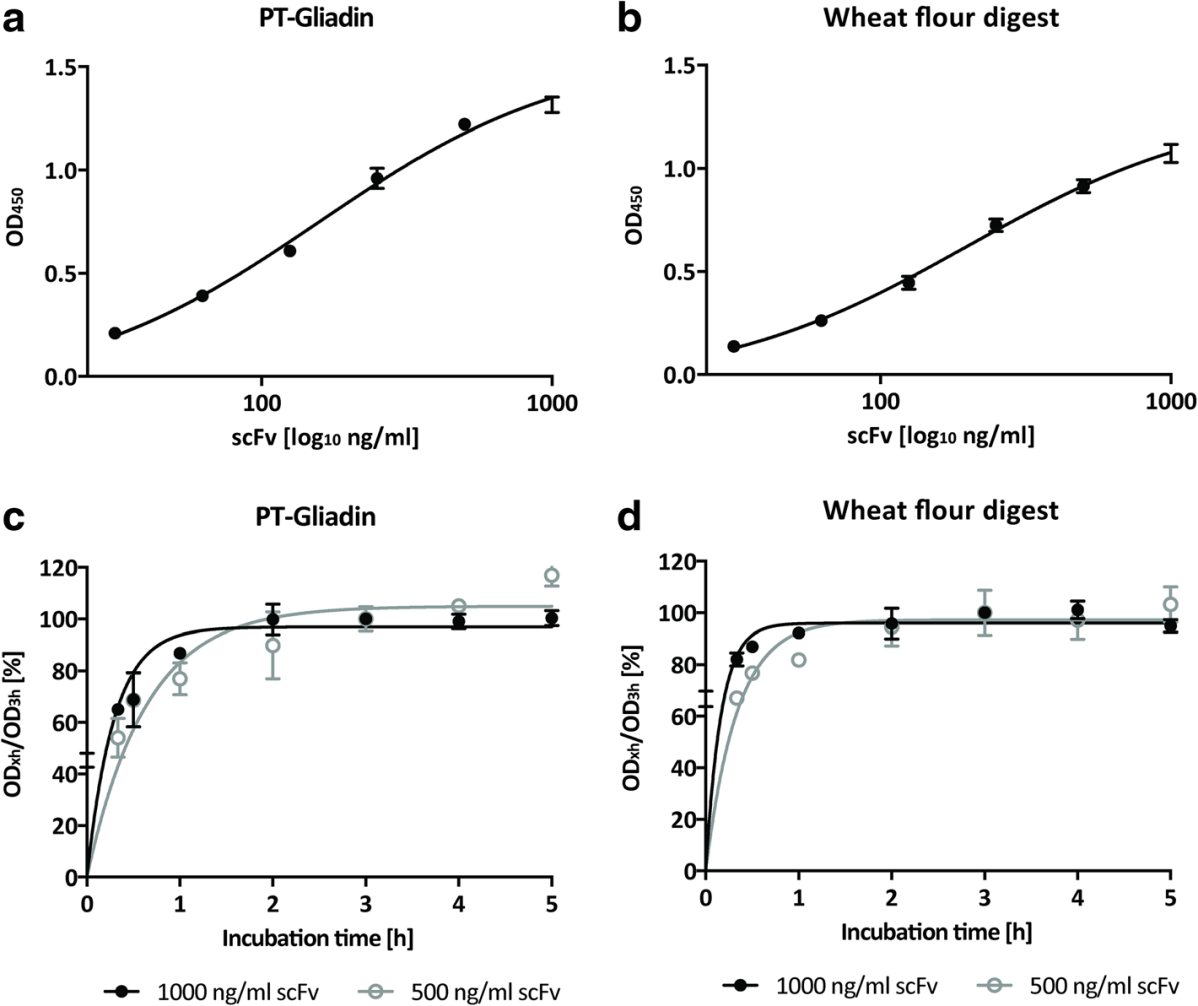
Purified scFv detects PT-Gliadin and natural wheat flour digest. **a**. ScFv binds to PT-Gliadin in a concentration -dependent manner. Purified scFv was diluted in indicated concentrations and binding was assessed by ELISA as described in Methods. Data shown are OD (450 nm) values and represent mean values (+/− SEM) of triplicates. **b**. Purified scFv binds to natural wheat flour digest in a concentration -dependent manner. Purified scFv was diluted in indicated concentrations and binding was assessed by ELISA as described in Methods. Data shown are OD (450 nm) values and represent mean values (+/− SEM) of triplicates. **c**. ScFv binding reaches approximate equilibrium after 3 h incubation time. Two single chain concentrations (500 and 1000 ng/ml respectively) were incubated for indicated time and binding to (**c**). PT-Gliadin or (**d**). wheat flour digest was assessed by ELISA as described in Methods. Data shown represent OD (450 nm) values at different incubation times compared to the OD (450 nm) value after 3 h (ODx/OD_3h_ *100). Mean values (+/− SEM) of triplicates are shown

**Fig. 7:**
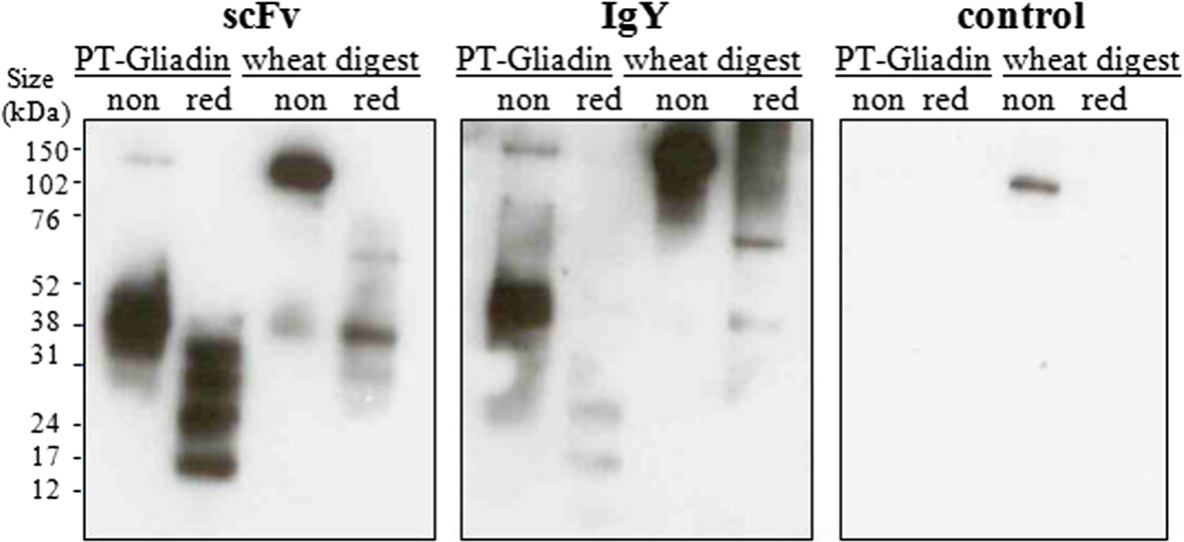
Purified scFv detects PT-Gliadin and natural wheat flour digest comparable to IgY. PT-Gliadin or wheat flour digest (10 μg per lane) were separated under reducing [red] or non-reducing [non] conditions on a 12 % Bis-Tris Gel and blotted on activated PVDF membranes. Western Blot was performed as described in Methods, using purified scFv in 1:500 (~15 μg/ml) or IgY in 1:100000 (~1 μg/ml) dilutions. Control represents a blot treated in the same way, but where primary antibody was omitted. Experiment shown is representative for at least three repeated experiments

ScFv candidate was purified to homogeneity by immobilized metal affinity and subsequent size-exclusion chromatography. Purified scFv showed concentration -dependent binding to PT-Gliadin as expected (Fig. 6a). Binding reached approximate equilibrium after 3 h of incubation time with scFv (Fig. 6c). We questioned whether scFv would also detect natural wheat antigens, mimicking the real-life situation. Common whole-wheat flour was subjected to gastric digestion in form of “simulated gastric fluid” (SGF), to resemble the protein portion that enters the upper intestinal duodenum after stomach passage. Gastric digested wheat flour, denoted as “wheat flour digest”, was coated onto 96-ELISA plates analogous to PT-Gliadin. Direct ELISA testing revealed concentration -dependent, specific binding of scFv (Fig. 6b), reaching approximate equilibrium after 3 h incubation time (Fig. 6d). Furthermore, scFv detects PT-Gliadin and wheat flour digest under non-reducing and reducing conditions in western blot, revealing similar binding patterns compared to IgY (Fig. 7).

### Polyclonal anti-PT-Gliadin-IgY competitively displaces scFv - evidence of a common epitope

**Fig. 8:**
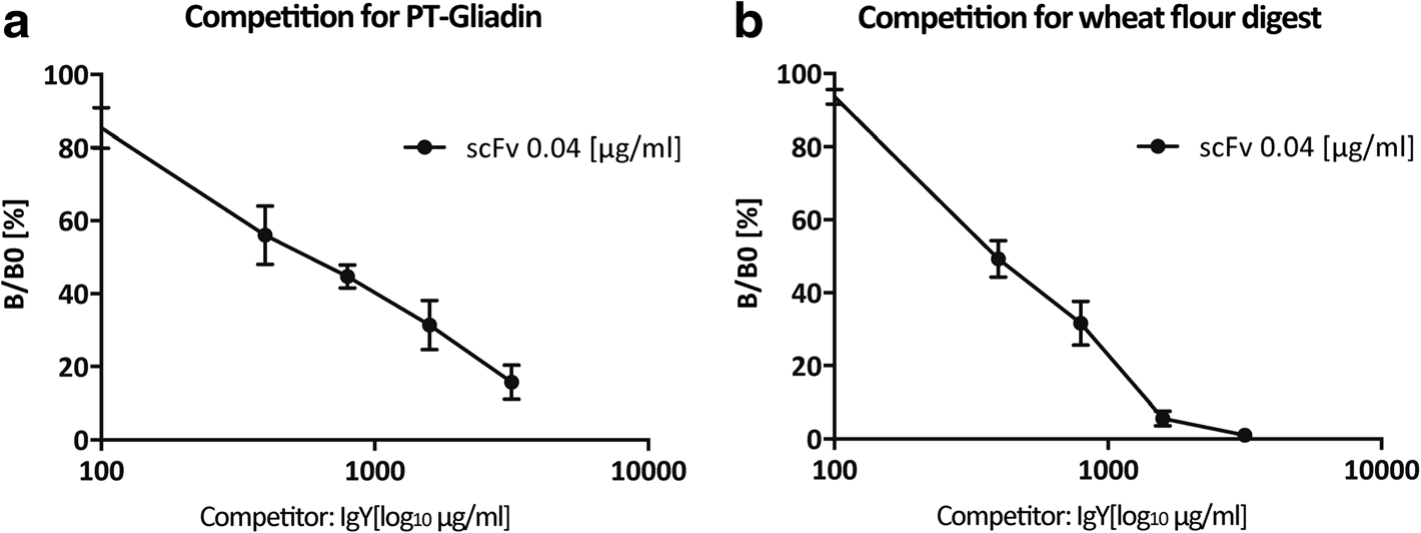
IgY competes with scFv for antigen binding sites. **a**. IgY competitively displaces scFv from PT-Gliadin coated plates. Competitive ELISA was performed with a 60 min pre-incubation with anti-PT-Gliadin IgY at indicated concentrations followed by addition of anti-PT-Gliadin-scFv at a concentration of ~ 0.04 [μg/ml] for 40 min. Detection was performed with anti penta-His antibody, as it detects specifically the His-tag labelled scFv but does not react with IgY. ELISA principle is described in Methods. Data shown are OD (450 nm) values and represent mean values (+/−SEM) of triplicates. **b**. IgY competitively displaces scFv from wheat flour digest coated plates. Competitive ELISA was performed as described in A and Methods. Data shown are OD (450 nm) values and represent mean values (+/−SEM) of triplicates

Since scFv exerts comparable binding properties to IgY, we hypothesized that they might have a common epitope. To address this theory, we developed a competitive ELISA assay, which allows specific detection of scFv via hexahistidine tag. For competition, IgY dilution range was pre-incubated for 60 min on coated antigen (PT-Gliadin or wheat flour digest, respectively). Then, scFv was added at constant concentration and bound scFv was detected by anti-penta-His antibody. IgY competitively displaces scFv to a high extent (86 % +/− 0.03 %) from PT-Gliadin coated plates (Fig. 8a) and almost fully (94 % +/− 1.14 %) displaces scFv from coated natural wheat flour (Fig. 8b). This data clearly argue for a common binding epitope between polyclonal IgY and monoclonal scFv, which is accessible in PT-Gliadin and in digested natural wheat flour in vitro.

### ScFv detects gluten-containing grain digests, including common wheat, khorasan wheat and rye

**Fig. 9:**
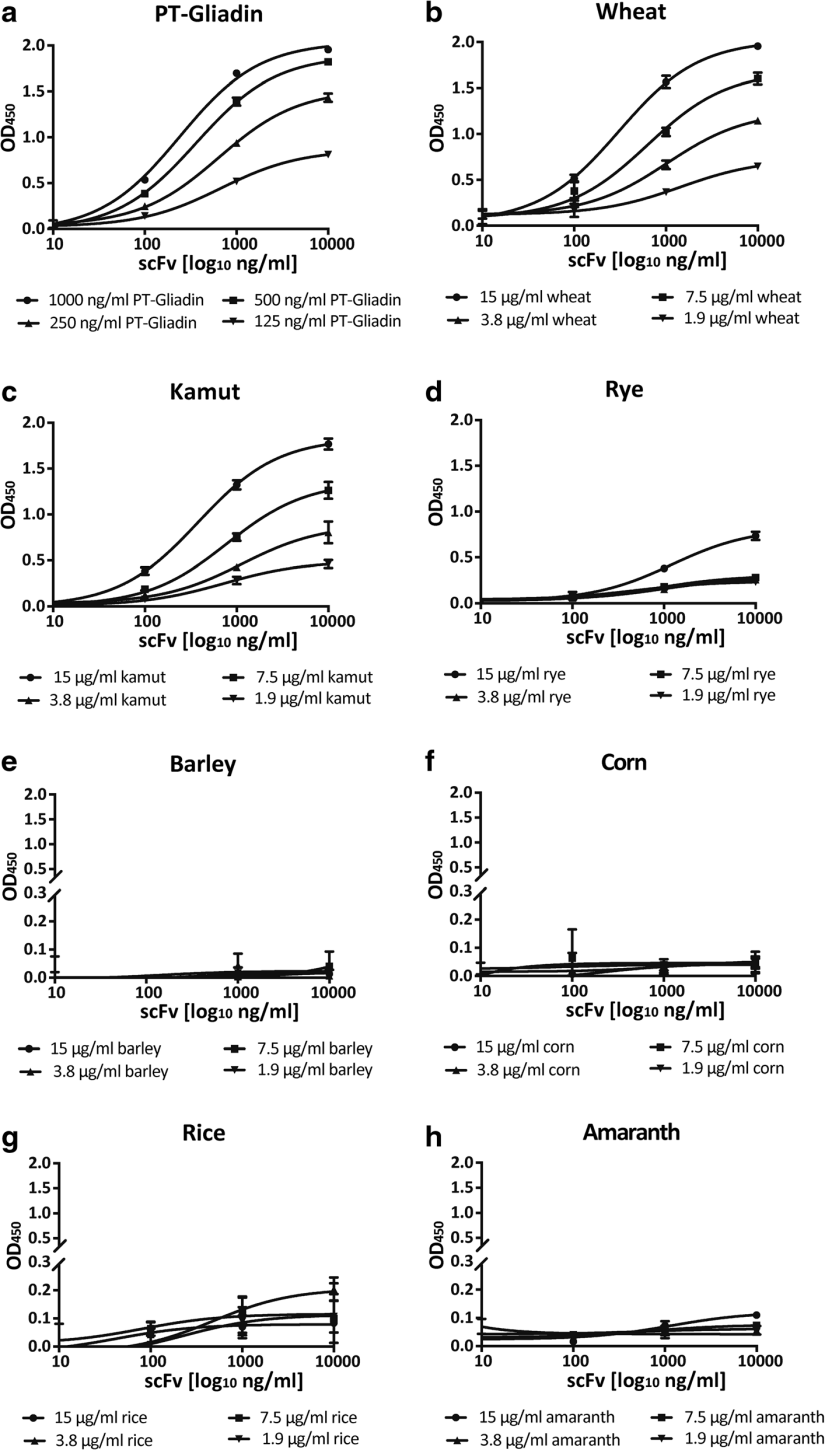
ScFv binds to digests of gluten-rich grains, but not to gluten-free grains or pseudo grains. ScFv was diluted to 10, 100, 1000 and 10,000 ng/ml and binding to various grain digests was assessed by ELISA as described in Methods. Data shown were analyzed by 3-parameter curve-fit nonlinear regression using Graph Pad Prism 6 software and are OD values (450 nm) representing the mean (+/− SEM) of triplicates. ScFv detects PT-Gliadin (**a**), common bread wheat (**b**), khorasan wheat (**c**) and to lower degree rye (**d**). ScFv does not react with barley (**e**), corn (**f**), or rice (**g**). ScFv does not detect the pseudo grain amaranth (**h**). Please note the axis break in **e, f, g** and **h**, which was introduced to demonstrate that OD_450_ does not exceed 0.2 and represents background signal

**Fig. 10:**
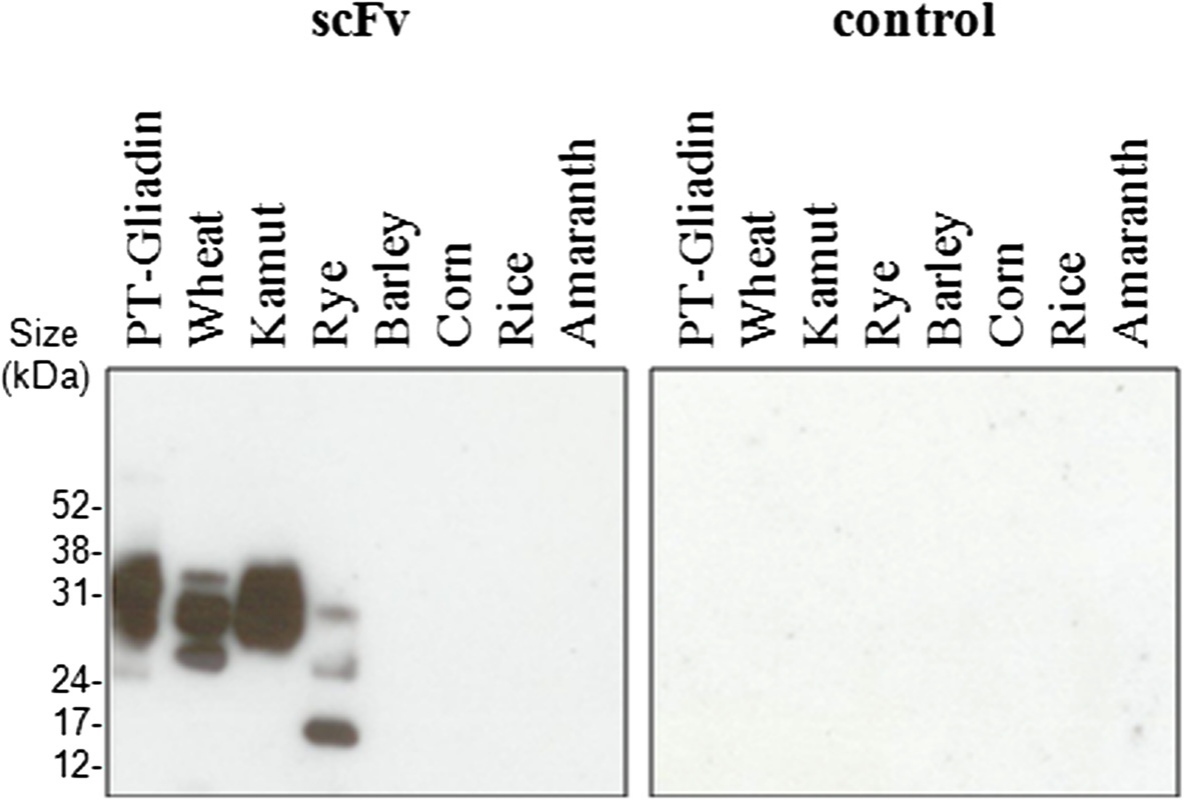
ScFv detects gluten-rich grain digests in Western Blot. PT-Gliadin (10 μg per lane) or different flour digests (50 μg per lane) were separated under reducing conditions on a 12 % Bis-Tris Gel and blotted on activated PVDF membranes. Western Blot was performed as described in Methods, using purified scFv in a 1:3000 (~2.5 μg/ml) dilution. Control represents a blot treated in the same way, but where primary antibody was omitted. Experiment shown is representative for at least two repeated experiments

As we had been able to demonstrate concentration -dependent, specific binding of scFv to PT-Gliadin and wheat flour digest, we wanted to assess scFv reactivity to other grains. Digests of common bread wheat, khorasan wheat, rye, barley, corn, rice and amaranth were coated in four protein concentrations onto 96-well ELISA plates. Four scFv concentrations in the range of 10–10000 ng/ml were subjected to the four antigen-coating concentrations by ELISA, and sigmoid binding curves were analyzed by Graph Pad Prism software. As kinetic binding experiments revealed that approximate equilibrium was reached after a 3 h incubation period with scFv (6 C and D), the incubation time for binding studies was adjusted to this incubation time. Sigmoid binding curves for PT-Gliadin, wheat digest and khorasan wheat digest were comparable (Fig. 9a, b, c), indicating that wheat prolamins are successfully targeted by scFv. Accordingly, scFv detected rye digest (Fig. 9d) to a lower extent than wheat digest. Notably, scFv did not react with barley (Fig. 9e), gluten-free grains like corn (Fig. 9f) and rice (Fig. 9g), or pseudo-grains like amaranth (Fig. 9h). ELISA data were further supported by western blot results, demonstrating scFv binding to PT-Gliadin, wheat flour digest, khorasan wheat flour digest and to a lesser degree to rye. On the contrary, scFv exerts no binding towards barley, corn, rice or amaranth (Fig. 10). Based on data of sigmoid binding curves, we wanted to calculate rough affinity constants for binding of scFv to PT-Gliadin and flour digests. For the calculation we relied on a method for the assessment of mean affinity constants (K_aff_mean) by direct ELISA, first described by Loomans et al. in 1995 [26]: Affinity constants were calculated based on the scFv concentration at OD_50_ of each sigmoid antigen curve. Four scFv concentrations subjected to four antigen-coating concentrations allow three affinity calculations for an antigen coating ratio of two and one for an antigen coating ratio of eight. Mean affinity constants, shown in Table 1, represent the mean of four calculated affinity calculations per antigen. ScFv targets PT-Gliadin, common and khorasan wheat digest with K_aff_ mean values in the high nanomolar range; 3.82 (+/−1.93) x 10^7^ M-^1^, 3.21 (+/− 2.14) x 10^7^ M-^1^ and 2.30 (+/− 1.49) x 10^7^ M-^1^; respectively.

**Table 1:** Equilibrium binding constants of scFv for PT-Gliadin and wheat digests

Antigen	K_aff_ mean (+/− SEM) [M^−1^]
PT-Gliadin	3.82 (+/− 1.93) x 10^7^
Wheat flour digest	3.21 (+/− 2.14) x 10^7^
Kamut flour digest	2.30 (+/− 1.49) x 10^7^

Data were analyzed by 3-parameter curve-fit nonlinear regression (Graph Pad Prism 6) as described in Methods. Mean affinity constants (+/− SEM) are shown

## Discussion

CD affects approximately 1 % of the population worldwide [3], with rising incidence in certain geographic areas explainable by environmental and life style factors [28].

Much research contributed in solving the puzzle of celiac disease, which is known today as a multi-factorial interplay of innate and adaptive immunity. In short, dietary derived prolamins termed ‘gluten’ break intestinal epithelial barrier and enter the *lamina propria,* where they provoke adverse reactions accomplished both by innate and adaptive immune cells. The only treatment option for CD remains the GFD which, though successful in most patients, is associated with substantial social burden, higher nutritional costs and compliance problems [29]. In some patients, however, even after long-term adherence to GFD, CD symptoms and clinical manifestations remain [30–32]. Overall, there is urgent need for therapeutic regimes complementing or replacing the GFD.

In this study, we present an avian scFv, selected from a chicken gene library, which targets PT-Gliadin and natural flour digests of wheat and rye. ScFv was expressed successful in soluble form in *E. coli*, purified to homogeneity and characterized by biochemical methods. We could demonstrate concentration -dependent specific binding of scFv to PT-Gliadin with binding affinities in the high nanomolar range. ScFv showed similar binding patterns to PT-Gliadin and wheat flour digest compared to polyclonal IgY, purified from yolks of immunized chicken. We designed a competitive ELISA experiment to demonstrate displacement of scFv by IgY, which argues for a common epitope between scFv and IgY. CD is provoked by dietary prolamins of the triticeaen grass tribe, including wheat gliadin, rye secalin and barley hordein. Strictly speaking, gliadin denotes the prolamin fraction of wheat. When referring to toxic celiac entities, however, the term gluten is used synonymously for noxious prolamins of different grain origin. By subjecting equal amounts of different whole grain flours to gastric digestion (by the aid of SGF, as mentioned in the experimental section), we were able to directly compare binding of scFv to different cereal grains high or devoid of gluten. ScFv shows comparable binding to common wheat flour and khorasan wheat flour digests. Khorasan wheat (*Triticum turgidum turanicum)*, traded as kamut, is considered an ancient relative of modern durum wheat (pasta wheat) [33]. Previously, it was assumed that ancient wheat strains like khorasan are devoid or low of celiac toxicity compared to modern industrial strains. Recent studies demonstrate the lack of difference between the toxicity of traditional and modern strains and recommend the total exclusion of all wheat varieties [33, 34]. Our observation, that scFv targets both, common bread wheat (*Triticum aestivum)* and khorasan wheat (*Triticum turgidum turanicum),* supports this view. *Triticum aestivum* is the predominantly used wheat species in the modern industrialized world, especially since its high protein content favors baking properties. It harbors the most immunogenic celiac toxins [35], though homologous toxic peptides can be found in rye and barley. Accordingly, scFv detected rye to a lesser extent than common or khorasan wheat. Notably, scFv failed to detect barley digest in our setting. Most studies assessing celiac toxicity are focusing on wheat gluten or digests thereof [36, 37], though immune reactivity to rye and barley was readily demonstrated in the past [38] and is reflected by the susceptibility of CD patients to these grain species. A recent study investigated significant differences in hordein spectra of different barley varieties, rendering malting barleys (*Hordeum vulgare*) less immunogenic than wild barleys (*Hordeum chilense)* [39]. In our experimental setting, six-rowed barley (*Hordeum vulgare hexastichon),* a representative of the *H. vulgare* species, was used. To our knowledge, degree of celiac toxicity has not been investigated explicitly for this barley subspecies so far. Overall, we assume that our scFv detects celiac noxious peptides present in substantial amounts in wheat and rye, but in quantities below the detection limit in barley. Another possibility is that the epitope targeted by scFv is present in wheat and rye digests, but absent or not accessible in barley digests. Rice and corn represent natural gluten-free grains and are essential part of the GFD [40]. Conclusively, scFv shows no reactivity towards these grain species. Recently, pseudo grains like amaranth, buckwheat and quinoa have entered the stage [41]. They resemble grains though they belong biologically to a distinct group, and therefore represent a welcome dietary alternative for CD patients. As expected, scFv showed no reactivity to amaranth digest. In summary, we were able to soluble express a scFv in *E. coli*, which exerts specific binding towards PT-Gliadin and gluten-containing wheat and rye digests, known to be noxious in terms of CD.

## Conclusions

We have successfully cloned and selected a scFv specific for noxious CD peptides. It has shown in vitro binding potential to PT-Gliadin and digests of gluten-containing grains, but exerts no reactivity to gluten-free grains and pseudo grains. We think that scFv can be of benefit for future CD treatment regimes.
